# Down‐regulated lncRNA HOTAIR alleviates polycystic ovaries syndrome in rats by reducing expression of insulin‐like growth factor 1 via microRNA‐130a

**DOI:** 10.1111/jcmm.14753

**Published:** 2019-11-16

**Authors:** Bin Jiang, Min Xue, Dabao Xu, Jiayu Song, Shujuan Zhu

**Affiliations:** ^1^ Department of Gynaecology The Third Xiangya Hospital of Central South University Changsha China

**Keywords:** Competitive binding, Endocrine, Granulosa cell, HOTAIR, Insulin‐like growth factor 1, MicroRNA‐130a, Polycystic ovaries syndrome, Targeted inhibition

## Abstract

It has been found that long noncoding RNA HOTAIR, microRNA‐130a (miR‐130a) and insulin‐like growth factor 1 (IGF1) expression are associated with ovarian cancer, thus, we hypothesised that the HOTAIR/miR‐130a/IGF1 axis might associate with endocrine disorders and biological behaviours of ovarian granulosa cells in rat models of polycystic ovary syndrome (PCOS). PCOS rat models were established by injection of dehydro‐isoandrosterone, followed by treatment of si‐HOTAIR, oe‐HOTAIR, miR‐130a mimics or miR‐130a inhibitors. Serum hormonal levels were determined to evaluate endocrine conditions. The effect of HOTAIR and miR‐130a on activities of isolated ovarian granulosa cells was assessed, as well as the involvement of IGF1.In the ovarian tissues and granulosa cells of PCOS rat models, highly expressed HOTAIR and IGF1 and poorly expressed miR‐130a were identified. In response to oe‐HOTAIR, serum levels of E_2_, T and LH were increased and serum levels of FSH were reduced; the proliferation of granulosa cells was reduced and apoptosis was promoted; notably, expression of miR‐130a was reduced while expression of IGF1 was increased. The treatment of si‐HOTAIR reversed the situation. Furthermore, the binding of HOTAIR to miR‐130a and targeting relationship of miR‐130a and IGF1 were confirmed. LncRNA HOTAIR up‐regulates the expression of IGF1 and aggravates the endocrine disorders and granulosa cell apoptosis through competitive binding to miR‐130a in rat models of PCOS. Based on our finding, we predict that competitive binding of HOTAIR to miR‐130a may act as a novel target for the molecular treatment of PCOS.

## 1 INTRODUCTION

Polycystic ovary syndrome (PCOS) is the most frequently occurring endocrine disorder in women during reproductive age, which is associated with hyperinsulinaemia, hyperandrogenaemia and aberrant adipokines production from the adipose tissue.^1^ PCOS is also a hormonal, metabolic and psychosocial disorder that severely impairs the patients’ quality of life.^2^ The incidence of PCOS is 6%‐10% according to the National Institute of Health criteria, which reaches 15% based on the broader Rotterdam criteria.^3^ PCOS is characterised by increasing ovarian stroma and antral follicles, in addition to ovarian cortical thickening and theca cell hyperplasia.^4^ Women with PCOS are highly vulnerable to anovulation and infertility, and even such risk factors (obesity, abnormal uterine bleeding and diabetes) contributing to the development of endometrial cancer.^5^ Multiple studies have provided evidence for the role of molecular targets in the treatment of PCOS, which include long noncoding RNAs (lncRNAs), microRNAs (miRNAs) and corresponding target genes.^6^, ^7^, ^8^

LncRNA profiles in granulosa cells from patients with PCOS and healthy women demonstrate that dysregulated lncRNAs may play significant roles in steroidogenesis and granulosa cell proliferation, shedding lights on the aetiology of PCOS.^9^ LncRNAs have emerged as important epigenetic mediators with critical functions in the development and disease.^10^, ^11^, ^12^ HOTAIR is a 2158 bp lncRNA localised to a boundary in the HOXC gene cluster.^13^ HOTAIR is highly expressed in primary breast tumours and the metastases, and the HOTAIR expression in primary tumours has been revealed to be a critical predictor of metastasis and death.^14^ For example, HOTAIR expression is associated with the metastasis of epithelial ovarian cancer and could serve as a prognostic biomarker and potential therapeutic target in epithelial ovarian cancer.^15^ In this study, we found that HOTAIR up‐regulated the expression of insulin‐like growth factor 1 (IGF1) via competitive binding to microRNA‐130a (miR‐130a). A study has pointed out that miR‐130a expression was associated with MDR1/P–glycoprotein‐mediated drug resistance in the ovarian cancer cells.^16^ IGF1 is a polypeptide hormone with high structural similarity to human pro‐insulin, and thus alterations of IGF1 activity has been proposed as an option in the treatment of endocrine disorders.^17^ Higher serum levels of IGF1 in PCOS women were previously reported to be correlated to the increased vascularity, which leads to the increased blood flow.^18^ Accordingly, we hypothesised that the expression of HOTAIR and miR‐130a might associate with the development of PCOS and their effects were exerted through regulating the expression of IGF1 in ovarian granulosa cells.

## 2 MATERIALS AND METHODS

### 2.1 Experimental animals

Totally, 90 Sprague Dawley (SD) rats (aged 21 d) were purchased from Beijing Vital River Laboratory Animal Technology Co., Ltd. Before being used in the experiments, the rats were allowed to adapt to the environment for 2 d. These rats were kept under controlled environmental conditions (22‐26°C; 12 hours light/dark cycle) with free access to food and water. The rats aged 23 day were classified into the normal group (n = 10) and PCOS group (n = 80). After model establishment, 10 PCOS rats were used to identify the modelling. After successful modelling, the rest 70 rat models of PCOS were assigned into 7 groups with 10 in each group: control group, EP group (injected with empty plasmid through the caudal vein), si‐HOTAIR group (injected with siRNA plasmid against HOTAIR through the caudal vein), oe‐HOTAIR group (injected with overexpression plasmid of HOTAIR through the caudal vein), negative control (NC) group (injected with NC of miR‐130a through the caudal vein), miR‐130a mimics group (injected with miR‐130a mimics through the caudal vein) and miR‐130a inhibitors group (injected with miR‐130a inhibitors through the caudal vein). The empty plasmid, siRNA plasmid against HOTAIR, overexpression plasmid of HOTAIR, NC of miR‐130a, miR‐130a mimics and miR‐130a inhibitors were purchased from Shanghai GenePharma Co., Ltd. (Pudong district, Shanghai, China). The animal experimental processes were approved by the Ethics Committee of The Third Xiangya Hospital of Central South University.

### 2.2 Model establishment and identification of PCOS

From the age of 23 days, PCOS rats were subcutaneous injected with dehydroepiandrosterone (DHEA) sulphate and sodium prasterone sulphate (9 mg/100 g bodyweight) and 0.4 mL water for injection, which lasted for 20 days. Rats in the normal group were injected with 0.4 mL water for injection at the same time point every day. At 20 days later, the rats were deprived of food for 12 hours. Then the ovaries were extracted. Through vaginal smear, the oestrous cycle was determined. Serum hormonal levels, haematoxylin‐eosin (HE) staining, transmission electron microscope (TEM) and terminal deoxynucleotidyl transferase‐mediated dUTP nick‐end labelling (TUNEL) assay were adopted to identify the models. When the models were determined as successful, the rats of each group were treated correspondingly the next day, and the continuous injection lasted for 7 days.

### 2.3 Enzyme‐linked immunosorbent assay (ELISA)

Rats of all the groups were deprived of food but not water after last injection. Following blood collection from heart, the serum was isolated. ELISA was employed to determine serum levels of follicle‐stimulating hormone (FSH), luteinising hormone (LH), teststerone (T) and oestradiol (E_2_) (Nanjing Jiancheng Bioengineering Institute, Nanjing, Jiangsu, China). The rats were euthanised by cervical dislocation, with both ovaries extracted. The morphology and colour of ovaries were observed. The weight was measured and recorded.

### 2.4 Haematoxylin and eosin staining

One side of the ovary was prepared and fixed in 10% formaldehyde for more than 24 hours. Then the ovary was dehydrated in gradient ethanol, permeabilised by xylene and embedded in paraffin after dipping in wax. The ovary was sliced to sections with a thickness of 4 μm. Subsequently, the sections were dewaxed and hydrated, followed by HE staining. After the treatment of gradient ethanol, the stained sections were permeabilised by xylene and mounted in neutral balsam. The morphological structures of ovarian follicles were observed under a light microscope.

### 2.5 Transmission electron microscope (TEM) to observe ultrastructure of ovarian follicles

One side of the ovary was prepared and fixed in 2% glutaraldehyde for 24 hours, followed by slicing into blocks (1 mm^3^). The ovarian blocks were immersed overnight after repeated rinsing by 0.1 mol/L phosphate‐buffered saline (PBS). Then the blocks were fixed in 1% osmic acid at 4°C for 2 hours, followed by washing by deionised water for three times (5 min per wash). At 4°C, the ovarian blocks were dehydrated in gradient acetone and embedded, followed by polymerising at 70°C for 36 hours. Next, the blocks were sliced to semi‐thin sections, which were stained by azure‐methylene blue and localised under a light microscope. Then, the sections were made to ultrathin sections, treated with double electron stain of uranyl acetate‐lead citrate. Under the TEM, the ultrastructures of ovarian granulosa cells, theca cells and lutein cells were observed and photographed.

### 2.6 TUNEL assay

The paraffin‐embedded ovarian tissues were collected for TUNEL staining. The number of apoptotic cells in the view field (×200) was calculated. Ten fields were counted and apoptotic cells in each field were recorded. The brown particles represented positive cells. The apoptotic index (AI) = (number of apoptotic cells/total cell number) × 100%.

### 2.7 Separation and culture of ovarian granulosa cells

The ovarian tissues of PCOS rats were collected and immersed in normal saline immediately. Under a microscope, the capsule of ovaries and surrounding adipose tissues were removed. Normal saline was used again to rinse the erythrocytes on the surface. The ovarian tissues were placed in the serum‐free DMEM/F12 medium (Gibco, Grand Island, NY, USA), where the follicles were pierced to release ovarian granulosa cells. Then, the ovarian granulosa cells were gently triturated to suspend single cells, which were filtered by a 200 mesh screen and centrifuged at 1000 rpm for 8 minutes with supernatant discarded and cells harvested. The deposited ovarian granulosa cells were added with DMEM/F12 medium supplemented with 15% foetal bovine serum (FBS) (Gibco, Grand Island) for incubation in a 5% CO_2_ incubator at 37°C. The medium was replaced 24 hours later when the cells adhered to the wall. After the adhered cells were removed, the mixture was further incubated. When the cell confluence reached 80%, the cells were detached, and the cells were harvested after centrifugation. The deposited granulosa cells were added with DMEM/F12 medium and triturated to prepare cell suspension, which were stained by trypan blue. The cells were counted under the microscope and then subcultured.

### 2.8 Cell grouping and treatment

The ovarian granulosa cells at logarithmic growth phase were grouped as follows: blank group (without any treatment), EP group (transfected with empty plasmid), si‐HOTAIR group (transfected with siRNA plasmid against HOTAIR), oe‐HOTAIR group (transfected with overexpression plasmid of HOTAIR), NC group (transfected with NC of miR‐130a) and miR‐130a mimics group (transfected with miR‐130a mimics). According to the instructions of lipofectamine 2000 (Invitrogen), the cells were transfected and incubated in the incubators for 48 hours, followed by subsequent experiments.

### 2.9 Dual‐luciferase reporter gene assay

The binding sites of lncRNA HOTAIR and miR‐130a were predicted by the bioinformatics website (https://cm.jefferson.edu/rna22/Interactive/). Next, binding relationship between lncRNA HOTAIR and miR‐130a was confirmed by using the dual‐luciferase reporter gene assay. The synthesised gene fragments of miR‐130a 3’UTR were inserted into the pMIR‐REPORT™ miRNA Expression Reporter Vector System (AM5795; Thermo Fisher Scientific, Massachusetts, USA) through such endonuclease sites as SpeI and Hind III. Complementary mutation sites were designed on the wild‐type (WT) miR‐130a. Through T4 DNA ligase, the target fragments (WT sequence and MUT sequence) were inserted into the pMIR‐REPORT™ miRNA Expression Reporter Vector System following restriction endonuclease cleavage. The right luciferase reporter plasmid WT and MUT after sequencing were cotransfected into the ovarian granulosa cells. After 48 hours of transfection, the 293T cells were harvested and lysed. The luciferase activity was evaluated by a dual‐luciferase reporter gene assay kit (Biovision) and a Glomax20/20 luminometer (Promega, Madison, WI, USA). The experiment was conducted for three times.

The bioinformatics website (http://www.targetscan.org) was searched to predict the targeting relationship between miR‐130a and IGF1 as well as the binding sites of miR‐130a and IGF1 mRNA 3’UTR. The IGF1 mRNA 3’UTR promoter region sequence containing miR‐130a binding sites were synthesised, and IGF1 3’UTR‐WT plasmid was constructed. On the basis of IGF1 3’UTR‐WT plasmid, binding sites were mutated to construct IGF1 3’UTR‐MUT plasmid, and the vector plasmid of IGF1 3’UTR‐WT plasmid and IGF1 3’UTR‐MUT plasmid were constructed by pMIR‐REPORT™ miRNA Expression Reporter Vector System (AM5795; Thermo Fisher Scientific). The experiment was performed according to the instructions of plasmid extraction kit (Promega). Cells in logarithmically growing were seeded in the 96‐well plate. When cell confluence reached 70%, cells were transfected according to instructions of lipofectamine 2000. IGF1 3’UTR‐WT plasmid and miR‐130a mimics plasmid were mixed and transfected into the ovarian granulosa cells. IGF1‐3’UTR‐WT + NC and IGF1‐3’UTR‐MUT + NC were, respectively, cotransfected with IGF1‐3’UTR‐MUT + miR‐130a mimics. After transfection of 48 hours, cells were harvested and lysed. The luciferase activity was detected by using a dual‐luciferase reporter gene assay kit. The experiment was conducted for three times.

### 2.10 RNA‐pull down

Cells were transfected with WT biotinylated miR‐130a and MUT biotinylated miR‐130a (50 nmol/L for each), respectively. After transfection of 48 hours, cells were harvested and washed by PBS and incubated for 10 minutes in specific cell lysate (Ambion). The sample cell lysate was collected separately (50 mL). Residual lysis was incubated with M‐280 streptavidin magnetic beads (Sigma, St. Louis, MO) coated with RNase‐free and yeast tRNA (Sigma) for 3 hours at 4°C. After washing in ice‐cold lysate two times, cells were rinsed in low‐salt buffer for three times and in high‐salt buffer once. Antagonist miR‐130a probe was set as a NC. Trizol method was adopted to extract total RNA. The expression of lncRNA HOTAIR was determined by reverse transcription‐quantitative polymerase chain reaction (RT‐qPCR).

### 2.11 RNA isolation and quantitation

Total RNA of tissues and cells was extracted by Trizol method (Invitrogen). The high quality of RNA was confirmed by ultra‐violet analysis and formaldehyde gel electrophoresis. Then 1 μg RNA was collected, which was reversely transcribed by avian myeloblastosis virus (AMV) to obtain cDNA. The primers of PCR were designed and synthesised by Shanghai GenePharma Co., Ltd. (Shanghai, China) (Table 1), with glyceraldehyde‐3‐phosphate dehydrogenase (GAPDH) or U6 as an internal reference. The amplification conditions were pre‐denaturation (94°C) for 5 minutes, denaturation (94°C) for 40 seconds, and followed by annealing for 40 seconds at 60°C, DNA strands extension for 1 minute at 72°C, this protocol ran for 40 cycles, followed by extension for 10 minutes at 72°C. The PCR products were verified by agarose gel electrophoresis. Threshold value was manually selected at the lowest point of all the parallelly rising logarithmic amplification curves, and the threshold cycle (Ct) value of each reaction tube was obtained. The data were analysed by 2^−ΔΔCt^, which represents the multiple proportions of target gene expression of the experimental group to the control group. The formula was: ΔΔCt = (ΔCt _target gene_ − ΔCt_reference gene_)_experimental group_ − (ΔCt _target gene_ − ΔCt_reference gene_)_control group_. The experiment was conducted for three times with mean value calculated.

**Table 1:** Primer sequences for RT‐qPCR

Gene	Sequence
HOTAIR	F: 5’‐CATGGATCCACATTCTGCCCTGATTTCCGGAACC‐3’
	R: 5’‐ACTCTCGAGCCACCACACACACACAACCTACAC‐3’
miR‐130a	F: 5’‐GCTCTTTTCACATTGTGCTACT‐3’
	R: 5’‐CAGTGCAATGTTAAAAGGGCATA‐3’
IGF1	F: 5’‐ATCTCTTCTACCTGGCACTCTG‐3’
	R: 5’‐GAAGCAACACTCATCCACAAT‐3’
GAPDH	F: 5’‐ACGGCAAGTTCAACGGCACAG‐3’
	R: 5’‐GACGCCAGTAGACTCCACGACA‐3’
U6	F: 5’‐CTCGCTTCGGCAGCACA‐3’
	R: 5’‐ACGCTTCACGAATTTGCGT‐3’

Abbreviations: F, forward; GAPDH, glyceraldehyde‐3‐phosphate dehydrogenase; IGF1, insulin like growth factor 1; miR‐130a, microRNA‐130a; R, reverse; RT‐qPCR, reverse transcription‐quantitative polymerase chain reaction.

### 2.12 Western blot analysis

The protein of tissues and cells were extracted, the protein concentration of which was examined according to the instructions of bicinchoninic acid (BCA) protein assay kit (Wuhan Boster Biological Technology Co., Ltd., Wuhan, Hubei, China). The extracted protein added with uploading buffer was boiled at 95°C for 10 minutes. The extracted protein was uploaded with 30 μg for each well. Next, the extracted proteins were separated by 10% polyacrylamide gel electrophoresis (Wuhan Boster Biological Technology Co., Ltd.), with electrophoresis voltage transferring from 80 to 120 V by wet transfer. Subsequently, the proteins were transferred onto polyvinylidene fluoride (PVDF) membranes with transferring voltage of 100 mV for 45‐70 minutes. The membranes were blocked with 5% bovine serum albumin (BSA) for 1 hour. Primary antibodies of IGF1 and β‐actin (1:1000; Abcam, Cambridge, MA, USA) were added and incubated at 4°C overnight, followed by washing three times (5 min per wash) in TBST. Corresponding secondary antibodies (Shanghai Miaotong Biotechnology Co., Ltd.) were added and incubated for 1 hour. The membranes were washed for three times (5 min per wash). Chemiluminescence reagents were employed to develop images. β‐actin was considered as an internal reference. The images of the gels were developed by the Bio‐Rad Gel Doc EZ Imager (Bio‐Rad, Hercules, CA, USA). The grey values of target protein bands were analysed by ImageJ software(National Institutes of Health, Bethesda, MA, USA). The experiment was conducted for three times with mean value calculated.

### 2.13 Cell counting kit‐8 (CCK‐8) assay

Cells of all the groups were collected. The former medium was discarded before detection. Each well was added with freshly prepared pre‐warmed mixture (100 μL) containing DMEM/F12 and 10% CCK‐8 (Beyotime Institute of Biotechnology, Dongjiang District, Shanghai, China). The mixture was added to the 96‐well plate by replacing the medium. At the same time, blank control well was set, which was only added with the mixture of DMEM/F12 and CCK‐8. The plate was incubated at 37°C. The optical density (OD) value of each group was measured with a wavelength of 450 nm at 24, 48 and 72 hours, respectively. The experiment was conducted for three times with mean value calculated.

### 2.14 Flow cytometry

Cells at logarithmic growth phase were detached in trypsin. Then cells were harvested after centrifugation, with supernatant discarded. Then cells were rinsed in pre‐cooled PBS at 4°C and centrifuged at 1000 rpm for 5 minutes, which was repeated two times. The cell density was adjusted to 10^6^ cells/mL. Subsequently, 200 μL cells were washed by 1 mL pre‐cooled PBS two times and centrifuged. Next, the cells were resuspended in 100 μL binding buffer, with 2 μL Annexin V‐FITC (20 μg/mL) added. Then cells were allowed to stand on the ice for 15 minutes without light exposure, followed by transferring to the flow tube. With 300 μL PBS added, each sample was also supplemented with 1 μL of PI (50 μg/mL) before uploading. The samples were detected within 30 minutes. With Annexin V as the X‐axis and PI as the Y‐axis, left upper quadrant represents mechanically injured cells, right upper quadrant represents late‐stage apoptotic cells or necrotic cells, left lower quadrant represents negative normal cells and right lower quadrant represents early apoptotic cells.

### 2.15 Statistical analysis

SPSS 21.0 (IBM Corp, Armonk, NY) was employed to analyse data in this study. Measurement data are expressed by mean ± standard deviation. Normally distributed measurement data between two groups were analysed by *t* test and comparison among multiple groups by one‐way analysis of variance. Pairwise comparison was conducted by the least significant difference t test. *P* < .05 was accepted as indicative of significant differences.

## 3 RESULTS

### 3.1 Rat model of PCOS is successfully established

From the 10 days following modelling, vaginal smear of rats was examined every day. Through the observation of 10 days, the rats in the normal group maintained regular oestrous cycle; whereas the rat models of PCOS had irregular oestrous cycle without ovulation.

**Figure 1:**
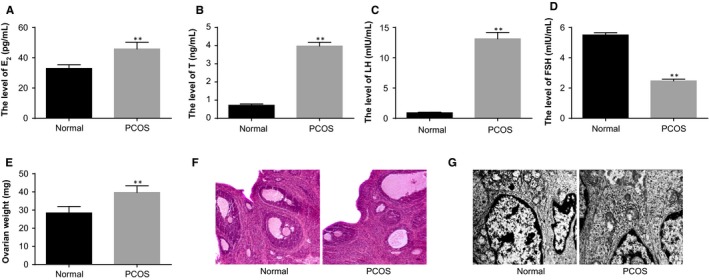
Identification of the establishment of rat models of PCOS. Note: A, serum levels of E_2_ in PCOS rat models and normal rats determined by ELISA; B, serum levels of T in PCOS rat models and normal rats determined by ELISA; C, serum levels of LH in PCOS rat models and normal rats determined by ELISA; D, serum levels of FSH in PCOS rat models and normal rats determined by ELISA; E, weight of ovaries of PCOS rat models and normal rats; F, HE staining to observe histopathological changes of ovarian tissues of PCOS rat models and normal rats (×100); G, the ultrastructures of ovaries of PCOS rat models and normal rats observed under a TEM (×10 000); **P < .01 compared with the normal group; PCOS, polycystic ovaries syndrome; ELISA, enzyme‐linked immunosorbent assay; E_2_, oestradiol; T, teststerone; LH, luteinising hormone; FSH, follicle‐stimulating hormone; and TEM, transmission electron microscope

The serum hormonal levels of rats were measured (Figure 1A‐D). Compared with the normal group, the serum levels of E_2_, T and LH were increased and serum levels of FSH were reduced in the PCOS group (all *P* < .05).

The general conditions of ovaries of rats were observed (Figure 1E). The rat ovaries in the normal group had red colour. In the PCOS group, the ovaries of rats were pale with increased size and saccular dilatation follicles observed on the surface. The weight of ovaries in the PCOS group was higher than that in the normal group (*P* < .05).

HE staining was used to observe the morphology of rat ovaries (Figure 1F). In the normal group, follicles of different developmental stages and many lutein cells were found on the cutting surface of ovaries; we also observed oocytes and corona radiata in mature follicles, uniformly aligned granulosa cells with intact morphology and thick cell layers (mostly 8‐9 layers). In the rat models of PCOS, plenty of saccular dilatation follicles were observed under the integumentumon the cutting surface of ovaries; the granulosa cells were loosely aligned, and the cell layers became thinner; proliferated thecal cells and a few lutein cells could be seen.

The ultrastructures of ovaries were observed under a TEM (Figure 1G). A number of mitochondria, granular endoplasmic reticulum, smooth endoplasmic reticulum, lipid droplets and microtubules were found in the normal group. In the PCOS group, the granulosa cells turned spindle‐shaped and the shape of nucleus was irregular; chromatin condensation inside the nucleus and intercellular fibrous proliferation could be seen; more layers of theca cells and lipid droplets in the thecal cells were observed. The aforementioned findings were highly suggestive of successful establishment of rat models of PCOS.

### 3.2 HOTAIR is highly expressed in ovarian tissues of PCOS rat models

**Figure 2:**
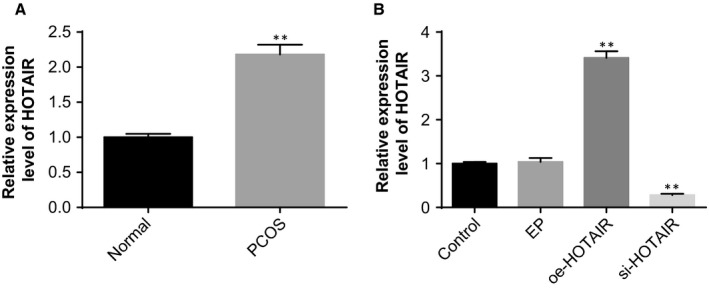
A high level of HOTAIR expression was identified in the ovarian tissues of PCOS rat models. Note: A, the expression of HOTAIR in the ovarian tissues of rats in the normal and PCOS groups detected by RT‐qPCR; B, the efficiency of interference to the HOTAIR expression was assessed by RT‐qPCR; **P < .01 compared with the normal group or control group; PCOS, polycystic ovaries syndrome; and RT‐qPCR, reverse transcription‐quantitative polymerase chain reaction

The expression of HOTAIR in the ovarian tissues of rats in the normal and PCOS groups was measured by using RT‐qPCR. PCOS rat models showed higher expression of HOTAIR than the normal rats (*P* < .05) (Figure 2A), which was indicative of the association of HOTAIR with PCOS.

The efficiency of interference to the HOTAIR expression in ovarian tissues was assessed by RT‐qPCR. Compared with the control and EP groups, the HOTAIR expression in ovarian tissues was significantly reduced in the si‐HOTAIR group and elevated in the oe‐HOTAIR group (both *P* < .05) (Figure 2B), suggesting successful interference to the HOTAIR expression in PCOS rat models.

### 3.3 HOTAIR accelerates endocrine disorders, ovarian injury and apoptosis of ovarian granulosa cells

**Figure 3:**
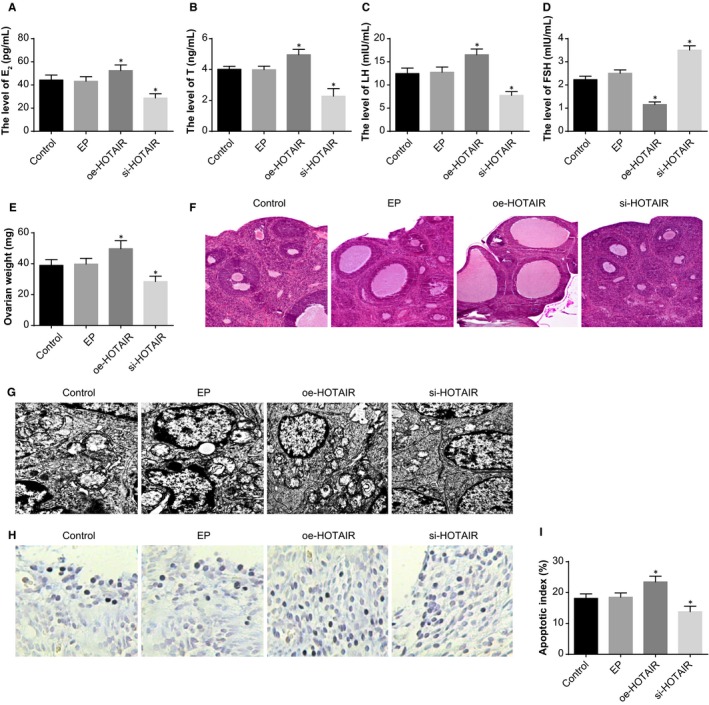
The effect of HOTAIR on the endocrine and ovarian granulosa cells in PCOS rat models. Note: A, serum levels of E_2_ in PCOS rat models determined by ELISA; B, serum levels of T in PCOS rat models determined by ELISA; C, serum levels of LH in PCOS rat models determined by ELISA; D, serum levels of FSH in PCOS rat models determined by ELISA; E, weight of ovaries of PCOS rat models; F, HE staining to observe histopathological changes of ovarian tissues of PCOS rat models (×100); G, the ultrastructures of ovaries of PCOS rat models observed under a TEM (×10 000); H, TUNEL staining to observe the apoptosis of ovarian granulosa cells in PCOS rat models (×200); I, quantitative analysis for the apoptotic index of ovarian granulosa cells in PCOS rat models; *P < .05 compared with the control group; PCOS, polycystic ovaries syndrome; ELISA, enzyme‐linked immunosorbent assay; E_2_, oestradiol; T, teststerone; LH, luteinising hormone; FSH, follicle‐stimulating hormone; TEM, transmission electron microscope; and TUNEL, terminal deoxynucleotidyl transferase‐mediated dUTP nick‐end labelling

In comparison to the control and EP groups, the serum levels of E_2_, T and LH were increased and serum levels of FSH were decreased in the oe‐HOTAIR group, whereas the situation was reversed in the si‐HOTAIR group (all *P* < .05) (Figure 3A‐D).

The weight of ovary in the oe‐HOTAIR group was higher and that in the si‐HOTAIR group was lower than that in the control and EP groups (*P* < .05) (Figure 3E).

HE staining (Figure 3F) was employed to observe the effect of oe‐HOTAIR and si‐HOTAIR on pathological changes of ovaries. In the control and EP groups, obviously saccular dilatation follicles and loosely aligned granulosa cells were observed; the layers of granulosa cells were reduced and the majority was 3‐4 layers; the thecal cells were proliferated and lutein cells were reduced. In the si‐HOTAIR group, no or little cystic dilatation was found in ovarian follicles; we also observed increased layers of granulosa cells, and oocytes and corona radiata in the dominant follicles. In the oe‐HOTAIR group, lutein cells and follicle development of different stages in the ovaries were decreased; few dominant follicles could be observed; reduced granulosa cells, atretic follicle and saccular dilatation follicles were observed.

Under a TEM, the ultrastructures of ovaries were observed (Figure 3G). In the control and EP groups, ovarian granulosa cells were irregularly shaped; plenty of round, oval or irregular mitochondria were found in the cytoplasm; some of the mitochondrial cristae were vacuolated; ribosome, granular endoplasmic reticulum and Golgi complex were abundant. In the oe‐HOTAIR group, plenty of mitochondria in round, oval or irregular shapes were observed in the granulosa cells; the mitochondrial cristae were vacuolated. In the si‐HOTAIR group, the cytoplasm of granulosa cells was abundant, in which a number of granular endoplasmic reticulum and smooth endoplasmic reticulum could be found; the mitochondria was reduced, most of which were round or oval shaped and a minority of which were in irregular shape; scattered lipid droplets and lysosomes were observed; the nuclei were in round or oblong shapes with uniform sizes.

TUNEL staining was used to evaluate the effect of HOTAIR on the apoptosis of ovarian granulosa cells (Figure 3H‐I). Compared with the control and EP groups, the AI of ovarian granulosa cells was increased in the oe‐HOTAIR group and decreased in the si‐HOTAIR group (*P* < .05).

### 3.4 HOTAIR inhibits proliferation and enhances apoptosis of ovarian granulosa cells

**Figure 4:**
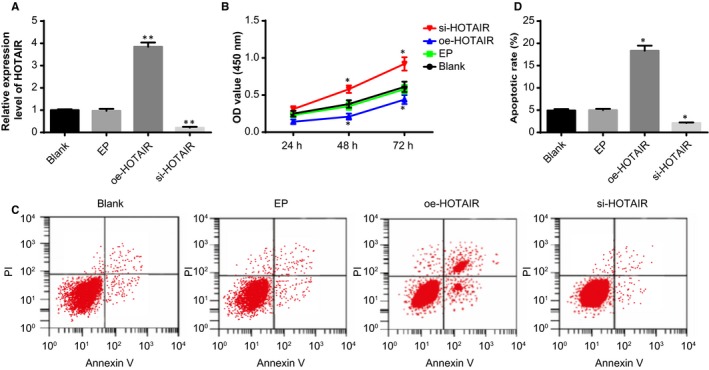
The effect of HOTAIR on the viability and apoptosis of ovarian granulosa cells in PCOS rat models. Note: A, the expression of HOTAIR in the ovarian granulosa cells detected by RT‐qPCR; B, viability of ovarian granulosa cells in response to oe‐HOTAIR and si‐HOTAIR detected by CCK‐8 assay; C, apoptosis of ovarian granulosa cells in response to oe‐HOTAIR and si‐HOTAIR detected by flow cytometry; D, quantitative analysis for the apoptosis rate of ovarian granulosa cells; **P < .01 compared with the blank group; *P < .05 compared with the blank group; PCOS, polycystic ovaries syndrome; RT‐qPCR, reverse transcription‐quantitative polymerase chain reaction; and CCK‐8, cell count kit‐8

The efficiency of interference to the HOTAIR expression in ovarian granulosa cells was assessed by RT‐qPCR. Compared with the blank and EP groups, the HOTAIR expression in ovarian granulosa cells was elevated in the oe‐HOTAIR group and reduced in the si‐HOTAIR group (*P* < .05) (Figure 4A), suggesting successful interference to the HOTAIR expression in ovarian granulosa cells of PCOS rat models.

The effect of HOTAIR on the viability of ovarian granulosa cells was examined by CCK‐8 assay (Figure 4B). No significant difference was found between the blank and EP groups in the viability of ovarian granulosa cells (*P* > .05). The viability of ovarian granulosa cells was increased in the si‐HOTAIR group and inhibited in the oe‐HOTAIR group, as compared with the blank and EP groups (*P* < .05).

The effect of HOTAIR on the apoptosis of ovarian granulosa cells was examined by flow cytometry (Figure 4C‐D). The apoptosis rate of ovarian granulosa cells was higher in the oe‐HOTAIR group and lower in si‐HOTAIR group than the blank and EP groups (*P* < .05). These results indicated that up‐regulated HOTAIR expression could suppress the proliferation and facilitate the apoptosis of ovarian granulosa cells.

### 3.5 HOTAIR inhibits expression of miR‐130a and promotes expression of IGF1 in ovarian tissues and granulosa cells

**Figure 5:**
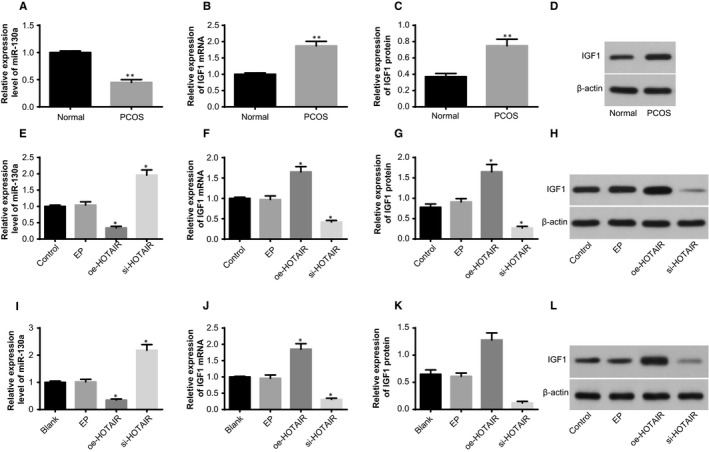
The effect of HOTAIR on the expression of miR‐130a and IGF1 in the ovarian tissues and granulosa cells. Note: A; the expression of miR‐130a in the ovarian tissues of rats in the normal rats and PCOS rat models; B, the mRNA expression of IGF1 in the ovarian tissues of rats in the normal rats and PCOS rat models; C, the protein expression of IGF1 in the ovarian tissues of rats in the normal rats and PCOS rat models; D, the protein band patterns of IGF1 in the ovarian tissues of rats in the normal rats and PCOS rat models; E, the expression of miR‐130a in the ovarian tissues of PCOS rat models; F, the mRNA expression of IGF1 in the ovarian tissues of PCOS rat models; G, the protein expression of IGF1 in the ovarian tissues of PCOS rat models; H, the protein band patterns of IGF1 in the ovarian tissues of PCOS rat models; I, the expression of miR‐130a in the ovarian granulosa cells of PCOS rat models; J, the mRNA expression of IGF1 in the ovarian granulosa cells of PCOS rat models; K, the protein expression of IGF1 in the ovarian granulosa cells of PCOS rat models; L, the protein band patterns of IGF1 in the ovarian granulosa cells of PCOS rat models; **P < .01 compared with the normal group; *P < .05 compared with the control group or blank group; and PCOS, polycystic ovaries syndrome

HOTAIR was expressed at a high level in ovarian tissues of PCOS rat models. According to the results of RT‐qPCR (Figure 5A‐D), lower expression of miR‐130a and higher expression of IGF1 were identified in the ovarian tissues of the PCOS rat models than the normal rats (*P* < .05), suggesting that HOTAIR may negatively regulate the expression of miR‐130a and positively regulate the expression of IGF1 in PCOS rat models.

The influence of HOTAIR on the expression of miR‐130a and IGF1 in PCOS rat models was evaluated by RT‐qPCR (Figure 5E‐H). Compared with the control and EP groups, the expression of miR‐130a was increased in the si‐HOTAIR group and decreased in the oe‐HOTAIR group; besides, the expression of IGF1 was reduced in the si‐HOTAIR group and elevated in the oe‐HOTAIR group (*P* < .05).

Furthermore, the effect of HOTAIR on the expression of miR‐130a and IGF1 in the ovarian granulosa cells was also determined (Figure 5I‐L). Compared with the blank and EP groups, the oe‐HOTAIR group showed reduced expression of miR‐130a and increased expression of IGF1; the si‐HOTAIR group displayed increased expression of miR‐130a and decreased expression of IGF1 (*P* < .05). The aforementioned findings implied that up‐regulated expression of HOTAIR could negatively regulate the expression of miR‐130a and positively regulate the expression of IGF1.

### 3.6 HOTAIR up‐regulates the expression of IGF1 via competitively binding to miR‐130a

**Figure 6:**
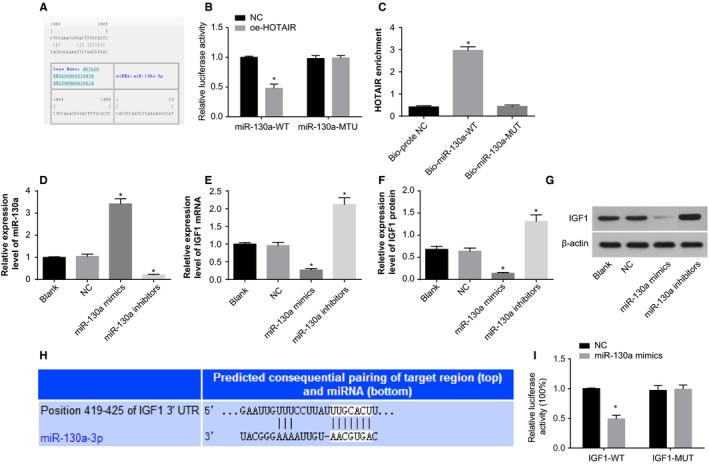
The identification of competitive binding of HOTAIR to miR‐130a as well as bindings of miR‐130a and IGF1. Note: A, the binding sites of HOTAIR to miR‐130a predicted by bioinformatic online software; B, the luciferase activity detected by dual‐luciferase reporter gene assay to verify the binding of HOTAIR to miR‐130a; C, the binding of HOTAIR to miR‐130a confirmed by RNA‐pull down assay; D, the expression of miR‐130a in the ovarian granulosa cells; E, the mRNA expression of IGF1 in the ovarian granulosa cells; F, the protein expression of IGF1 in the ovarian granulosa cells; G, the protein band patterns of IGF1 in the ovarian granulosa cells; H, the binding sites of miR‐130a to IGF1 predicted by bioinformatic online software; I, the luciferase activity detected by dual‐luciferase reporter gene assay to verify the targeting relationship of miR‐130a to IGF1; *P < .05 compared with the NC group or blank group; PCOS, polycystic ovaries syndrome; NC, negative control

Bioinformatics online software analysis demonstrated specific binding region of HOTAIR gene sequence to miR‐130a sequence (Figure 6A). Dual‐luciferase reporter gene assay was employed to confirm the binding relationship of HOTAIR to miR‐130a (Figure 6B). Compared with the NC group, the luciferase activity in the WT oe‐HOTAIR group was reduced (*P* < .05), and no significant difference was identified in the MUT luciferase activity (*P* > .05), suggesting the binding of HOTAIR to miR‐130a.

RNA‐pull down assay results (Figure 6C) indicated that, compared with the Bio‐probe NC group, the expression of HOTAIR in the Bio‐miR‐130a‐WT group was significantly increased (*P* < .05); besides, no difference was determined in the Bio‐miR‐130a‐MUT group (*P* > .05). These results implied that Bio‐miR‐130a‐WT could promote the enrichment of HOTAIR around it, following which, the competitive binding of HOTAIR to miR‐130a reduced the dissociation of miR‐130a.

In comparison with the blank and NC groups, the expression of miR‐130a was increased in the miR‐130a mimics group and decreased in the miR‐130a inhibitors group; moreover, the mRNA and protein expression of IGF1 was decreased in the miR‐130a mimics group and increased in the miR‐130a inhibitors group (*P* < .05) (Figure 6D‐G). Thus, we could conclude that the interference to the expression of miR‐130a in the ovarian granulosa cells was successful and miR‐130a negatively regulated the expression of IGF1.

Bioinformatics online software (http://www.targetscan.org) predicted the targeting relationship of miR‐130a and IGF1 (Figure 6H). The IGF1‐3'UTR‐WT plasmid and miR‐130a mimics were cotransfected in the ovary granulosa cells. In the WT, the luciferase activity in the IGF1‐3'UTR‐WT + NC group was lower than that in the IGF1‐3'UTR‐WT + miR‐130a mimics group (*P* < .05). In the MUT, the luciferase activity in the IGF1‐3'UTR‐MUT + NC group didn't differ from that in the IGF1‐3'UTR‐MUT + miR‐130a mimics group (*P* > .05) (Figure 6I). The aforementioned results indicated that HOTAIR repressed the inhibitory effect of miR‐130a on IGF1 and increased the expression of IGF1 by competitive binding to miR‐130a.

### 3.7 MiR‐130a attenuates endocrine disorders, ovarian injury and apoptosis of ovarian granulosa cells

**Figure 7:**
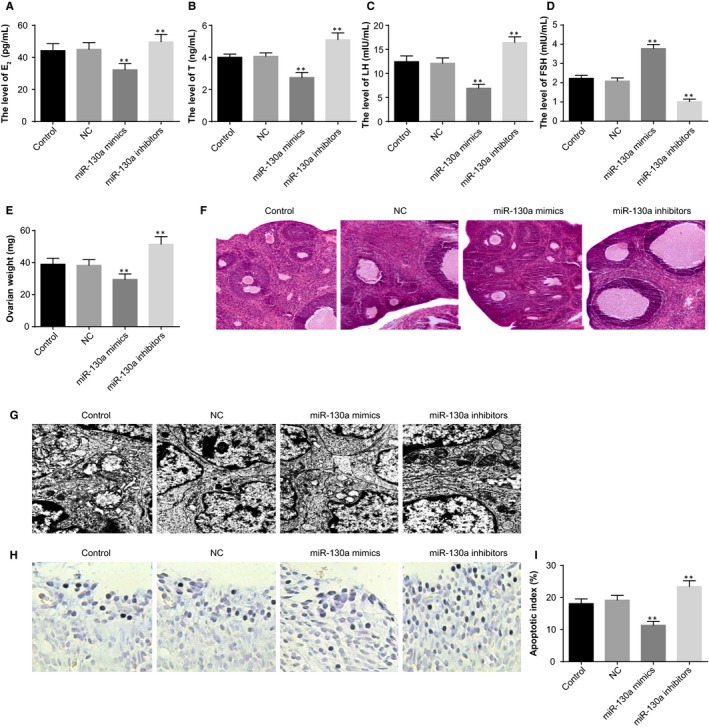
The effect of miR‐130a on the endocrine and ovarian granulosa cells in PCOS rat models. Note: A, serum levels of E_2_ in PCOS rat models determined by ELISA; B, serum levels of T in PCOS rat models determined by ELISA; C, serum levels of LH in PCOS rat models determined by ELISA; D, serum levels of FSH in PCOS rat models determined by ELISA; E, weight of ovaries of PCOS rat models; F, HE staining to observe histopathological changes of ovarian tissues of PCOS rat models (×100); G, the ultrastructures of ovaries of PCOS rat models observed under a TEM (×10 000); H, TUNEL staining to observe the apoptosis of ovarian granulosa cells in PCOS rat models (×200); I, quantitative analysis for the apoptotic index of ovarian granulosa cells in PCOS rat models; **P < .01 compared with the control group; PCOS, polycystic ovaries syndrome; ELISA, enzyme‐linked immunosorbent assay; E_2_, oestradiol; T, teststerone; LH, luteinising hormone; FSH, follicle‐stimulating hormone; TEM, transmission electron microscope; and TUNEL, terminal deoxynucleotidyl transferase‐mediated dUTP nick‐end labelling

The serum levels of E_2_, T and LH were not significantly different between the control group and NC group (all *P* > .05). Compared with the control and NC groups, serum levels of E_2_, T and LH were reduced and serum levels of FSH were elevated in the miR‐130a mimics group, whereas the situation was reversed in the miR‐130a inhibitors group (*P* < .05; Figure 7A‐D).

The weight of ovary in the miR‐130a mimics group was lower and that in the miR‐130a inhibitors group was higher, when compared with the control and NC groups (*P* < .05; Figure 7E).

Under the light microscope, in the control and NC groups, obviously cystically dilatated follicles and loosely aligned granulosa cells were observed; the layers of granulosa cells were reduced and few lutein cells could be found. In the miR‐130a mimics group, cystic dilatation in ovarian follicles was reduced and layers of granulosa cells were increased. In the miR‐130a inhibitors group, plenty of saccular dilatation follicles could be seen; lutein cells and follicle development of different stages in the ovaries were decreased; few dominant follicles and granulosa cells could be observed (Figure 7F).

The ultrastructures of ovaries were observed under a TEM (Figure 7G). In the control and NC groups, ovarian granulosa cells were irregularly shaped; plenty of round, oval or irregular mitochondria and vacuolated mitochondrial cristae were found in the cytoplasm. In the miR‐130a inhibitors group, large numbers of round, oval or irregular mitochondria and obviously vacuolated mitochondrial cristae were observed in the ovarian granulosa cells. In the miR‐130a mimics group, a number of granular endoplasmic reticulum and smooth endoplasmic reticulum could be found in the ovarian granulosa cells; the mitochondria was reduced, most of which were round or oval shaped; almost no lipid droplets and few lysosomes were observed; the nuclei were in round shape with uniform sizes.

TUNEL staining was used to evaluate the effect of miR‐130a on the apoptosis of ovarian granulosa cells (Figure 7H‐I). The AI of ovarian granulosa cells was lower in the miR‐130a mimics group and higher in the miR‐130a inhibitors group than the control and NC groups (*P* < .05).

### 3.8 MiR‐130a enhances proliferation and inhibits apoptosis of ovarian granulosa cells

**Figure 8:**
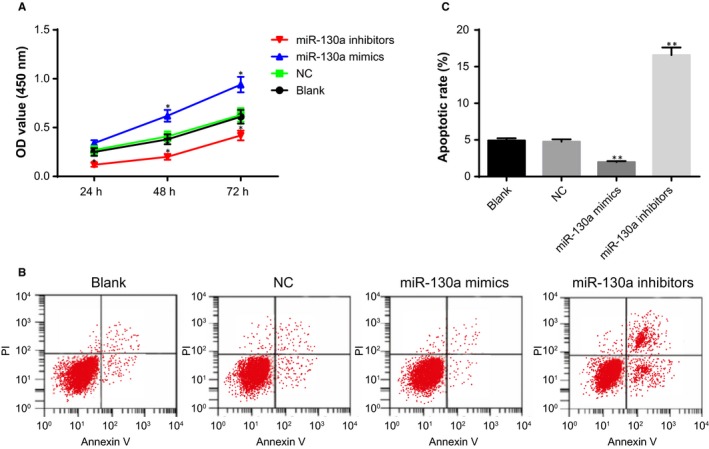
The effect of miR‐130a on the viability and apoptosis of ovarian granulosa cells in PCOS rat models. Note: A, viability of ovarian granulosa cells in response to miR‐130a mimics and miR‐130a inhibitors detected by CCK‐8 assay; B, apoptosis of ovarian granulosa cells in response to miR‐130a mimics and miR‐130a inhibitors detected by flow cytometry; C, quantitative analysis for the apoptosis rate of ovarian granulosa cells; **P < .01 compared with the blank group; *P < .05 compared with the blank group; PCOS, polycystic ovaries syndrome; and CCK‐8, cell count kit‐8

The effect of miR‐130a on the viability of ovarian granulosa cells was examined by CCK‐8 assay (Figure 8A). Increased viability and proliferation rate of ovarian granulosa cells was found in the miR‐130a mimics group; reduced  viability and proliferation rate of ovarian granulosa cells was identified in the miR‐130a inhibitors group, as compared with the blank and NC groups (*P* < .05).

The effect of miR‐130a on the apoptosis of ovarian granulosa cells was examined by flow cytometry (Figure 8A). The apoptosis rate of ovarian granulosa cells was lower in the miR‐130a mimics group and higher in miR‐130a inhibitors group than the blank and NC groups (*P* < .05). These results indicated that up‐regulated miR‐130a expression could promote the proliferation and impede the apoptosis of ovarian granulosa cells.

## 4 DISCUSSION

PCOS, as the most common cause of female infertility across the globe, is a multi‐causal and genetically complicated disorder, which is implicated in the failure in endocrine glands.^19^, ^20^ In the ovarian cancer, inhibited HOTAIR expression in ovarian cancer cells could impede the tumourgenesis and metastasis.^21^, ^22^ According to Deng *et* al, HOTAIR modulates the self‐renewal, growth, tumour metastatic and formation of the cancer stem‐like cell subpopulation enriched from breast cancer cells.^23^ Moreover, the levels of IGF1 are elevated and may affect ovarian function and increase androgen production in PCOS.^24^ Herein, we identified the expression of HOTAIR, miR‐130a and IGF1 in the ovarian tissues and granulosa cells of PCOS rat models and verified the regulatory relationships among them, so as to determine the mechanisms of controlling the endocrine disorders and activities of ovarian granulosa cells.

PCOS rat models were established by injection of DHEA. In the separated ovarian tissues and granulosa cells of rat models of PCOS, a high level of HOTAIR expression and IGF1 expression as well as a low level of miR‐130a expression were identified. It has been proved that HOTAIR rs920778 polymorphism is associated with ovarian cancer susceptibility and prognosis in a Chinese population.^25^ Then, the therapeutic value of HOTAIR in ovarian and breast cancers has been demonstrated using tumour specific peptides inhibits HOTAIR activity.^26^ Silencing of HOTAIR could inhibit the tumour growth and increase chemosensitivity of ovarian tumours in nude mice through regulation of HOXA7.^27^ In this present study, we found that HOTAIR accelerated the endocrine disorders, ovarian injury and apoptosis of granulosa cells in rat models of PCOS. HOTAIR is located between HoxC11 and HoxC12 in the human genome and mediates HoxD expression in multiple tissues.^28^ A study has revealed that IGF1 expression was elevated in human epithelial ovarian cancer samples in relation to that in benign ovarian tumour samples.^29^ Another study also proved that IGF1 level was up‐regulated in plasma of well‐differentiated epithelial ovarian cancer.^30^ As Zhang *et* al state that miR‐130a expression was markedly reduced in cisplatin‐resistant ovarian cancer cells.^31^ Epigenetic alterations of HOX genes can be correlated with PCOS and consequently female infertility, which provide insight for novel treatments with epidrugs for this disease. Notably, HOTAIR was validated to negatively regulate the expression of miR‐130a and positively regulate the expression of IGF1 in PCOS rat models. Furthermore, we confirmed that HOTAIR repressed the inhibitory effect of miR‐130a on IGF1 and increased the expression of IGF1 by competitive binding to miR‐130a.

It has been suggested that miR‐130 expression interacting with Hox genes could control vascular morphogenesis in developing lung.^32^ The role of miR‐130a was characterised in reducing HOXA5 expression, thus decreasing p53 expression and controlling breast cancer cells resulting in tumour progression and metastasis.^33^ MiR‐130a attenuated endocrine disorders, ovarian injury and apoptosis of granulosa cells in rat models of PCOS. The expression of miR‐130a has been examined in ovarian cancer cells, and it is involved in the cell activities and drug resistance.^16^ MiR‐130a may be a potential treatment target in ovarian cancers through inhibiting PTEN to activate PI3K/AKT signalling pathway.^34^ According to a previous integrated gene network analysis, miR‐130a expression is associated with multidrug resistance in epithelial ovarian cancer by binding to NRP1.^35^ MiR‐130a enhanced proliferation and inhibits apoptosis of ovarian granulosa cells in the rat models of PCOS of this study. MiR‐130a directly targeted and negatively regulated the expression of IGF1. When the expression of IGF1 was reduced, women with PCOS may be more sensitive to the treatment of octreotide.^36^ IGF1 might be attributed to the increase in serum levels of LH and the consequent hyperandrogenic anovulation in women with PCOS.^37^, ^38^ The bioavailability of IGF1 has been reported to play a key role in oocyte maturation in PCOS patients.^39^

By demonstrating that HOTAIR up‐regulated the expression of IGF1 via competitive binding to miR‐130a in the rat models of PCOS, we provide insight into the mechanisms underlying the promotion effect of up‐regulated HOTAIR expression in the endocrine disorders and granulosa cell apoptosis. We show that HOTAIR up‐regulates the expression of IGF1 and aggravates the endocrine disorders and granulosa cell apoptosis through competitive binding to miR‐130a in rat models of PCOS. Based on our finding, we predict that competitive binding of HOTAIR to miR‐130a may act as a novel target for the molecular treatment of PCOS. However, whether treatment will affect other organs and systems and whether it will directly or indirectly affect the ovaries will be explained in future study.

## CONFLICT OF INTEREST

The authors declare that they have no conflicts of interest.

## AUTHORS' CONTRIBUTIONS

Bin Jiang served as guarantor of integrity of the entire study. Shujuan Zhu designed the study. Min Xue and Dabao Xu contributed to the literature research. Jiayu Song contributed to the experimental studies.

## ETHICS STATEMENT

The animal experimental processes were approved by the Ethnic Committee of The Third Xiangya Hospital of Central South University.
